# South West Paediatric Club—Meeting at Barnstaple, May 1989

**Published:** 1990-03

**Authors:** Frederic Brimblecombe

**Affiliations:** Honorary Professor of Child Health, Postgraduate Medical School, University of Exeter


### South West Paediatric Club

### Meeting held at Barnstaple, May 1989

### Aspects of the History of Child Health in South West England. Victor Neale Lecture 1989

**Frederic Brimblecombe, CBE, MD, FRCP**
Honorary Professor of Child Health,
Postgraduate Medical School, University of Exeter

### INTRODUCTION
It is not only an honour but a labour of love to give a lecture decidated to Victor Neale. He retired in 1965, a quarter of a century ago, so that the numbers of us who worked with him when he was in practice are beginning to diminish. I think if I had to sum up in a single word what I regard as Victor Neale's outstanding quality it would be *caring*. He cared about everyone and everything – sometimes impulsively, often passionately. When very junior and very inexperienced consultants like me found themselves in desperate need of advice, he was the person we turned to. That I was in Exeter and he in Bristol made no difference, he never failed us. It is a joy that Phyllis is here to share our memory of him.

### INFECTIOUS DISEASE

#### Typhoid
I am going to describe a few fragments of the history of the health of children in the far west of England and in Devon in particular. I start by quoting from a paper in the Lancet of 1856. It is by William Budd, initially a general practitioner in North Tawton in Mid-Devon and later a consultant physician in Bristol. It is on typhoid fever.

'As the disease is by far the most fatal to persons in the prime of life, the father or mother or both, are the first to perish when death ensues, and the young survivors being left without support, their home is broken up and their destitution becomes complete. How often have I not seen in past days, in the single narrow chamber of the workman's cottage, the father lying in the coffin, the mother in the sick bed in muttering delirium, and nothing to relieve the desolation of the children but the devotion of some poor neighbour, who too often paid the penalty of her kindness in becoming herself the victim of the same disorder .... From the fact already referred to, of its being so much more deadly to grown-up persons, this disease has a relation to pauperism which is peculiar to itself. In this fever the workhouse finds its chief purveyor .... One of the most interesting of the many writers who have dwelt on this point expresses himself in the following words "A fever which consigns thousands to the grave consigns tens of thousands to a worse fate, for fever cuts off the parents, leaving the wretched offspring to fill the future ranks of prostitution, mendicacy and crime".'

William Budd's work in Devon and then in Bristol was carried out more than 40 years before the typhoid bacillus was identified. Even so his observations of the epidemic which affected 80 people in North Tawton enabled him to describe the clinical and epidemiological differences between typhoid and typhus fever. He confirmed that typhoid was spread by contagion. Later in Bristol when typhoid broke out in Richmond Terrace, Clifton in 1859, he demonstrated that typhoid might be a water borne disease. There were then 34 houses in Richmond Terrace of which 13 drew their water from one common pump, which was not used by the other houses. Typhoid broke out in every one of these 13 houses but in none of the other 21 houses.

William Budd was the son of Dr Samuel Budd, general practitioner in North Tawton. Through them began a six generation line of doctors, if one includes a daughter Eva who married a Dr Harper, who practiced in North Devon. The present generation is, of course, represented by Peter Harper, genetecist in Cardiff and John, paediatrician in Northampton. I went to mention my gratitude to their father Richard, who was senior physician at the North Devon Infirmary when I was first appointed in 1954. He was kindness itself and a great support to me as the first paediatrician on the staff of the North Devon Infirmary. There is a story of perinatal paediatrics about him. The winter of 1947 was one of the worst on record. In the February of that winter, Dick Harper's telephone rang in his house in Boutport Street at 2 a.m. A 16 year old girl was in labour on Exmoor, in a cottage somewhere near Blackmore Gate. Would he come? Dick and his partner Dr Smart set off by car on the icy roads to the rendezvous at Blackmore Gate. There, two horses and a guide were waiting to take them to the cottage 6 miles distant. Dr Smart put on his skis. Dick looked at the two horses and decided one looked a bit tamer than the other. The midder bags were strapped to the wilder one for the guide to lead and Dick mounted the more docile one.

The snow drifts were deep, the road unidentifiable. The wild horse reared up and bolted, the midder bags clanking as it disappeared. They found the horse buried in a snow drift with only its nose showing. It was rescued, but the midder bags had broken open. The forcep blades were retrieved but the traction handle was missing.

After 3 hours they found the cottage. The baby was born and bonny, but they were just in time to deliver the after birth. They then had to wait for daylight, when they said 'Goodby' to the new mother. 'Cheerio' she said, but never a word of thanks. They got home just in time to start the days surgery.

To return to enteric fever, William Budd's is not the only scientific contribution from Devon on this subject. In 1946, Dr Brendan Moore, Director of the PHLS in Exeter was asked to investigate an outbreak of paratyphoid in Woolacombe. His studies were obstructed by the Town Council who were afraid of adverse publicity to the their tourist trade. Fortunately in another outbreak in Sidmouth the authorities were more cooperative and as a result the Moore sewage swab became established, to become the internationally accepted technique in the epidemiological detection of enteric fever. This is described in Dr Moore's account of a recurrence of paratyphoid in Woolacombe by which time the local council had become a bit more anxious and a bit more humble. Using his special sewage swab he isolated paratyphoid organisms from the sewage outfall on the foreshore at Woolacombe. Then by taking swabs from each manhole from the sewage pipes back from the outfall and eliminating those which were negative, he arrived at the sewage from a single house in Woolacombe in which paratyphoid was present. This was inhabited by the wife of the ice cream vendor whose kiosk was well known on the beach at Woolacombe. She had all along been the unsuspecting symptomless paratypoid carrier of Woolacombe, which through the ice creams had infected many children of holiday makers. I commend to you Dr Brendan Moore's paper 'On Intestinal Fever – 125 years on' which was published in 1987. In that paper he describes the work not only of William Budd, but of another Devon physician, Sir Henry Acland FRS, knighted in his own right and also a son of the tenth baronet of Killerton, became Regius Professor of Medicine at Oxford. He wrote extensively about cholera forecasting the identification and pathogenecity of specific bacteria in infectous disease long before the time of Thomas Koch and Pasteur.

**Cholera**

In connection with cholera I must refer to Dr Thomas Shapter, Physician to the Devon and Exeter Hospital who published his history of the Exeter cholera epidemic in 1832, one of the first in western Europe. He describes 400 cases in which the case mortality was identical at all ages from infancy, through adult life and in the over 65s – an interesting contrast to the relative mildness of typhoid in children as described by William Budd.

**Measles**

If I devote time to infectious disease, it reminds us that the child population of the West of England like the rest of the country was repeatedly devastated by lethal epidemics comparable to those in Third World countries today. Haemorrhagic measles for example, as in Africa now, was a principal killer. I cannot leave the history of infectious disease without reference to smallpox.

**Smallpox**

It is interesting that the 17th century descriptions of the disease like William Budd's acount of typhoid again state that smallpox was a milder disease in children than in adults. This is mentioned for example by a Dorsetshire man the famous Dr Thomas Sydenham. It is appropriate to spend a moment on this remarkable man remembered for his descriptions of Sydenham's chorea, measles and scarlet fever. Dewhurst (1966) in his biography of Sydenham describes how as a convinced Parliamentarian he had little time for medicine until he was 37 being called upon to serve as a combatant soldier in a regiment of Foot raised for the Parliamentarians by his brother in Dorchester. During the Civil War the Royalists had Sydenham's father locked up in Exeter jail but his sons William and Thomas contrived his release. In fact Thomas Sydenham eventually did very well out of the war financially since Cromwell appointed him to a sinecure but highly lucrative job as 'Commissioner of Free Farm Rents'. This enabled him to set up in practice in a fashionable part of London. Later when his fame as a physician established him as the leading clinician in the country, it was with some discretion that he attended members of the Court and household of Charles II. He was only in active practice for 16 years, becoming a semi-invalid by the age of 53.

At the end of the century smallpox caused the death of Queen Mary (of William and Mary) in 1694. This was followed by the great epidemic of 1710 in London. By this time the disease had become as lethal to children as to adults. Although variolation against smallpox had already been used in Constantinople for 50 years, it was not until the early years of the 18th century that it was introduced into England. Variolation using the fluid from vesicles of patients with the disease was to be a matter of major medical controversy for the rest of the 18th century.

In a multicentre trial in 1726 involving London, Norwich, Winchester, Exeter and Plymouth, 256 people were so treated with 4 deaths. In 1743 Dr Dod of Bridport reported severe reactions in children he inoculated and to his dismay despite these reactions these same children two years later contracted severe smallpox when an epidemic affected the town. Some of you visited Ebford Manor the house near Exeter where Esther and I lived for 25 years until I retired. In 1748 it was owned by Matthew Lee. In his diary of that year he wrote 'On October 19th Matty was taken ill with sickness and fever. On the 21st smallpox began to appear, but fortunately in a mild form and through the goodness of God he was perfectly recovered in 14 days. We took Jenny to Mr Huckell's house as soon as Matty was taken ill. On October 28th she was vaccinated with the fluid from Matty's pox. She began to sicken on the 4th of November and on the 6th the smallpox appeared and continued for several days. It pleased the Almighty that the operation should succeed, Jenny did not have in all more than 50 spots on her whole body. To our surprise some days after she was brought home her legs began to swell and pimples appeared. This proved to be a fresh crop of smallpox pustules ripening gradually until they had white heads, then dried up and the swelling of her legs subsided. After this she was perfectly recovered'.

Before concluding my references to smallpox I want to point out that in the mid 18th century, Devon with its medical school in Exeter, probably had the most distinguished physicians ever to practice in the county in the whole of its medical history. These included Dr John Huxham of Plymouth, who was the first to describe paresis of the palate as a complication of diphtheria. Dr Huxham like his three contemporaries in Exeter, Thomas Glass, Michael Lee Dicker and Dr John Andrew had all been pupils of Boerhaave in Leyden. It is interesting to see how the commercial links with the low countries over the wool trade had complemented academic links in medicine. Thomas Sydenham was regarded as the outstanding physician of the seventeenth century. Boerhaave of Leyden, who was greatly influenced by Sydenham, occupied a similar status in the eighteenth century. Sir George Baker, later PRCP, a Devonian, returned to Exeter to identify the lead in the cider presses as the cause of the Devonshire colic.

The controversy about smallpox variolation continued and there is a masterly monograph on this subject published in 1765 by Dr Andrew of Exeter. "The Practice of Inoculation Impartially considered". Dr John Mudge of Plymouth in 1776 used a preparation heavily diluted with water which many doctors regarded as safe but totally ineffective. It was not until 1798 that Dr Edward Jenner's famous work in Berkely established the value of vaccinia vaccination in place of variolation. Even so there was much suspicion and its use was far from universally applied. Through much of the 19th century those working in the tourist trade in Bath had to try on several occasions to hush up publicity about epidemics. There was a major epidemic in Bristol in 1840 in which many children were affected.

### HOSPITALS

The first record of a hospital in Devon is of the one founded by Leofric, a Cornishman, after his appointment as Bishop of Exeter in 1046. The hospital was at Callendarhay (now in the grounds of the Bishop's Palace adjoining the cathedral). It was amalgamated in 1225 with another hospital which became

**Admissions to Devon and Exeter Hospital 1753**

(Total Admissions 1225. Deaths 64)

| *Admission diagnosis* | *Age 0–5* | *5–9* | *10–14* | *Total* |
|---|---|---|---|---|
| Trauma | 4 | 14 | 14 | 32 |
| Scrofula | 2 | 2 | 7 | 11 |
| Skin rash | 2 | 1 | 3 | 6 |
| Eye inflammation | 1 | 5 | 2 | 8 |
| Abscess | 1 | 3 | 3 | 7 |
| Urinary calculus | 2 | 2 | 2 | 6 |
| Convulsion | 1 | – | 4 | 5 |
| Hernia | 2 | 2 | 1 | 5 |
| Respiratory infection | – | – | 2 | 2 |
| Chorea | – | 1 | 1 | 2 |
| Rheumatic Fever | – | 1 | – | 1 |
| Scurvy | – | 1 | – | 1 |
| Miscellaneous | 1 | 7 | 7 | 15 |
| Total | 16 | 39 | 46 | 101 |

known as St John's Hospital. In 1327 Bishop Grandison enlarged the hospital and added a school for orphans to its resources. In 1620 the school became a Grammar School known as the Blue Coats School, but the hospital was diminishing in importance. In 1876, the original foundation of St John was divided into three separate organisations, Heles School for boys, Maynard School for girls and Wynards as a hospice for the elderly.

The mid 18th century saw the establishment of the major provincial voluntary hospitals, Winchester and Bristol in 1736, York 1740, Exeter 1741 and Bath 1742. The Plymouth Dispensary was established in 1789 and its hospital in 1835. Truro's first hospital was opened in 1799.

I have reviewed the in-patient Admission Register for the Devon and Exeter Hospital for 1753, 10 years after the hospital had come into full operation. There were in all 1224 admissions with 64 deaths. Of the admissions 101 were children under the age of 15. Clearly the admissions were highly selected to exclude infectious disease and of course the usual practice, except among the poor, was to treat sick children in their own homes. No special wards were set aside for children. In Truro a curious rule was made, namely that it would be 'improper' to admit any children or pregnant women to the hospital. This lasted for 50 years from 1799 to 1850. Elsewhere in the mid nineteenth century many cities initiated the practice of separating children from adults either by building children's hospitals as in Bristol or by establishing children's wards in general hospitals; this was done in 1860 in Exeter when Sir John Bowring gave the necessary endowment to make this possible. It was not until 65 years later in 1925 that Truro established a children's ward in its hospital.

**Royal Western Counties Hospital**

I think the mid 19th century must have been a very exciting time for anyone concerned with promoting child health and welfare. In Devon there was much concern about handicapped children and especially the mentally retarded. I am indebted to David Gladstone of the Department of Social Administration of Exeter University for help with this section of my story.

In 1864 there was a meeting in Exeter chaired by the Earl of Devon 'To establish an institution for idiot children who appear likely by careful treatment to be capable of mental improvement'. This lead to the formation of the Royal Western Counties Hospital at Starcross.

It was believed that by withdrawing these children from their own homes and treating them in an institution *by suitable training* they would be capable of learning skills which would provide them with employment. The age range for such training was strictly limited to 6–15 years. If there was no improvement with training the child was returned home or to the Poor Law workhuse. In any case all children were discharged at the age of 15.

For admission they had:

(1) To be assessed by the medical officer as suitable for training.
(2) Their progress would be reviewed and if they failed to improve with training, they were discharged.
(3) Epileptics and those defined as insane were excuded.

The training programmes were intensive and varied – shoe making and repairing, carpentry, baking, gardening and farm labouring, lace and dress making, housework leading to employment as housemaids and kitchen maids to mention only a few. In a labour intensive market this policy proved highly successful There was much competition for places first from the West of England and then from as far away as London and Birmingham. Funding was through the Poor Law or the Local Authorities via their Education and Mental Deficiency Committees.

Sadly 40 years later by 1900 this enlightened policy was beginning to change. Society wished the mentally hadicapped to be segregated and hidden away in segregated institutions for life. This was reflected in the Mental Deficiency Act of 1913 which laid down that a person could be placed in an institution if *defective* (a very different concept to that on which the Western Counties Hospital had been founded, namely suitability for *training*). The categories for admission were:

(1) Being defective or abandoned.
(2) Guilty of a criminal offence.
(3) Habitual drunkard.
(4) Moral defective.

There was no limitation on the age of admission or the length of the stay. In the words of Roy Parker, Starcross changed from a flowing stream into a stagnant pond. The emphasis was altered to one of custodial care although valiant efforts to continue the training role were made.

One needs also to mention the disastrous results of the advent of psychologists – lead by Binet in 1908 who first introduced intelligence testing. One of my great friends is Yuri Bronfenbrenner, Professor of Human Development at Cornell. His father was a neuropathologist to a similar hospital in the USA. It was Bronfenbrenner senior's delight to meet the new children admitted and coach them in how to improve their score on Binet's I.Q. tests. He coud then send them back to their Education Authorities saying they had been wrongly assessed and to the amazement of the psychologists who on re-testing could only confirm that their ability to solve the tests was now vastly improved and admission to an institution was not justified.

### SOME MORE RECENT HISTORY

When I was appointed in 1954 as the first paediatrician in Exeter with responsibility also for Torbay and North Devon the only paediatrician west of me was Hugh Jolly, at Plymouth, who had been appointed in 1952. I started work on the 1st January. It was immediately apparent that infectous gastro-enteritis was rampant in the children's ward in Exeter. A review of the records showed that during the previous 2 months 26 infants admitted to the ward for conditions other than diarrhoea (such as infantile eczema and pyloric stenosis) had contracted gastro-enteritis as a result of admission to the ward and 6 of them had died as a result. Within 2 weeks of starting work I reported to the Group Secretary (General Manager) that the children's ward must have a proper milk kitchen and proper cubicled isolation facilities for all infants. 'Doctor' he said 'It would be a waste of time to draw up plans; there is no money for such a scheme'. To cut the story short, these facilities were functioning 4 weeks later as a result of a little help from publicity in the Daily Express and the BBC.

There was no such thing as a SCBU. Pre-term infants were nursed in the only nursery attached to the maternity ward and by the midwives who were also responsible for the deliveries. There was only 1 trained midwife on duty at night and if we had a sick pre-term infant with recurrent apnoea it had to be left whilst the midwife attended new mothers admitted in labour. It took about 18 months to establish a SCBU and that only by commandeering the bedroom allocated to the paediatric house officer and consigning him to sleep in the attic of the hospital along with the housemaids. In his bedroom we installed incubators, but most importantly midwives and nurses whose sole duty it was to nurse infants and who had no responsibility in the labour wards. It was a great privilege that when the opportunity to design our first purpose built SCBU occurred (which was opened in 1959, 5 years after my initial appointment), Dr Vicky Crosse from the Sorrento Hospital came to Exeter to advise on its design. When Dr Jaco the first paediatrician in Truro introduced an incubator into the nursery in the maternity unit in the early 1960s it was thrown down the stairs and smashed by the consultant obstetrician.

Visiting in children's wards was for 1 hour a week on Sunday afternoons. The battle with the nurses and with the surgeons (particularly orthopaedic surgeons) to achieve unrestricted visiting has been long and bitter, but is being won more by attrition than a single *coup de main*!

There was an isolation hospital in Exeter, presided over by a Doctor who was a caricature of a Prussian Officer. He felt it his duty to keep the wards full, thus if a child was admitted he would say in addition to its presenting illness that it had primary tuberculosis and had to stay in for 18 months or 2 years. He allowed no parental visiting at all. Very occasionally these children would become unwell and with great reluctance he would call me in to see them. It was at this point that his Achilles heel was revealed. He could not resist a good tonsillectomy. This enabled me to establish my Scarlet Pimpernel escape network. By advising tonsillectomy, the child was transferred to the general hospital, where my friend the ENT surgeon and I would agree that the operation was unnecessary and the child would be discharged home. This was as far as I know the only escape route from the Stalag Isolation Hospital. The Prussian doctor was so thick that I don't think he ever realised what was happening.

It was less easy to do much about the other long sentence children; that is those with spinal and joint tuberculosis admitted to the orthopaedic hospital for periods of 2 to 3 years.

Fortunately the prevention of bovine tuberculosis resolved this problem without intervention from paediatricians.

Finally one of the most rewarding forms of paediatrics was to do between 250–300 domiciliary consultations a year. Rewarding not only financially but much more for other reasons. It came about because not having a registrar, the G.Ps would ring me up for advice about a sick child, unlike registrars of subsequent times, I could often say 'Do you think if I saw the child at home with you, we could avoid hospital admission?'. On many occasions this proved to be possible and many children with Henoch Schonlein Purpura, acute nephritis, glandular fever, and many others were successfully nursed at home rather than undergo admission to hospital. It also meant that the partnership between general practitioners and paediatricians was close and I have no doubt beneficial to both as well as to the children.

### REFERENCES

ANDREWS, C. T. (1975) 'The First Cornish Hospital'. Wordens Ltd: Penzance, Cornwall.

ANDREW, JOHN (1767) 'The Practice of Inoculation impartially considered' in 'New Observations on Inoculation'. Maty, M., Brussels.

BOOTH, SIR CHRISTOPHER (1989) Herman Boerhaave and the British *J. Royal Coll. Physicians Lond.* **23**, 194.

BUDD, WILLIAM (1856) On intestinal fever: its mode of propagation. *Lancet* 694.

CREIGHTON, C. (1894) 'A History of Epidemics in Britain'. Cambridge: Cambridge University Press.

DELPRATT HARRIS, J. (1922) 'The Royal Devon & Exeter Hospital'. Eland: Exeter.

DEWHURST, KENNETH (1966) 'Dr Thomas Sydenham'. Wellcome Historical Medical Library.

GLADSTONE, D. E. (1989) 'Reconstructing the Past: Issues in the Historical Study of Institutional Care'. Paper presented to South Bank Polytechnic, London.

HARPER, MARGERY (1983) 'Dr Richard Harper of Barnstaple'. Pitman Press: Bath.

MOORE, BRENDAN (1987) On intestinal fever – 125 years on. *Trans. Devon Ass. Advant: Sci.* **119**, 1–15.

RUSSELL, P. M. G. (1976) 'A History of the Exeter Hospitals'. Exeter Postgraduate Medical Centre.

SHAPTER, THOMAS (1832) 'History of the Cholera in Exeter'. Churchill: London.

### AN OUTBRAK OF PULMONARY TUBERCULOSIS IN PRE-SCHOOL CHILDREN

**A. R. J. Bosley, M. George and G. George**
North Devon District Hospital, Barnstaple

Pulmonary Tuberculosis was diagnosed in a 4 year old boy with a four week respiratory illness. Intensive contact tracing in his small market town in North Devon identified 28 children with radiological tuberculosis. Attendance at one of two children's Christmas parties was a common factor to all. The source case was a mother with open pulmonary tuberculosis, herself a contact 13 years previously. Most of the children were of pre-school age. 43 (3.8%) out of 1139 children screened were Heaf positive.

Radiological changes seen were: miliary tuberculosis, 2; hilar lymphadenopathy, 22; Ghon foci, 2; tuberculous pneumonia, 2; subsequent lobar collapse and symptoms secondary to hilar lymphadenopathy, 4. Chemotherapy was complicated by the limited range of palatable preparations for young children. All patients showed minor drug side-effects but only one child exhibited drug intolerance.

No cases of active pulmonary disease were seen in older contacts who had received BCG previously. A major problem was the limited choice of anti-tuberculous drugs marketed in a presentation suitable for young children.

Methodical contact tracing and record keeping, plus the cooperation of local medical services, identified a major outbreak of tuberculosis in young children and contained media interest to an informative level. This outbreak illustrates that tuberculosis can be of high virulence and infectivity in susceptible individuals with minimal exposure.

### PERSPECTIVES OF ANOREXIA NERVOSA

**Dr Scilla M. White**
North Devon District Hospital, Barnstaple

In many ways anorexia is a problem of control. Firstly the patient controls appetite, food intake and weight. Later this extends to control of those around, particularly family. In treatment this issue of control has to be addressed from the start and a clear statement about who is in control of what is made.

Parents should have control over the decision whether treatment is to be undertaken or not. In order to take this decision they need information about timing and venue for treatment, consequences of not treating, or interrupting treatment once started, and the likely pressures on parents and family and the support available to them.

The anorexic is given control over whether they cooperate with the regime or not. Rewards are given for cooperation (i.e. not cheating or exercising, eating what is asked of them) and not for weight gain. The aim is to regain normal eating habits starting with very small portions chosen from the normal menu, backed up half an hour after the meal by an enriched milk drink. Rewards and the timing of increased food and activity are determined by the clinician in negotiation with nursing staff, parents and patient. Control later passes from clinician to parents.

